# Web-based survey investigating cardiovascular complications in hypermobile Ehlers-Danlos syndrome after COVID-19 infection and vaccination

**DOI:** 10.1371/journal.pone.0298272

**Published:** 2024-03-21

**Authors:** Anthony L. Guerrerio, Allyson Mateja, Gretchen MacCarrick, Jonathan Fintzi, Erica Brittain, Pamela A. Frischmeyer-Guerrerio, Harry C. Dietz

**Affiliations:** 1 Division of Gastroenterology, Hepatology and Nutrition, Department of Pediatrics, Johns Hopkins University School of Medicine, Baltimore, Maryland, United States of America; 2 Clinical Monitoring Research Program Directorate, Frederick National Laboratory for Cancer Research, Frederick, Maryland, United States of America; 3 McKusick-Nathans Institute of Genetic Medicine, Johns Hopkins University School of Medicine, Baltimore, Maryland, United States of America; 4 Biostatistics Research Branch, National Institute of Allergy and Infectious Diseases, National Institutes of Health, Bethesda, Maryland, United States of America; 5 The Laboratory of Allergic Diseases, National Institutes of Allergy and Infectious Diseases, National Institutes of Health, Bethesda, Maryland, United States of America; 6 Howard Hughes Medical Institute, Chevy Chase, Maryland, United States of America; Institute of Theoretical and Applied Informatics Polish Academy of Sciences: Instytut Informatyki Teoretycznej i Stosowanej Polskiej Akademii Nauk, UKRAINE

## Abstract

**Background:**

Hypermobile Ehlers-Danlos syndrome is a heritable connective tissue disorder associated with generalized joint hypermobility but also other multisystem comorbidities, many of which may be exacerbated during a viral illness or after a vaccination. We sought to determine whether individuals with hypermobile Ehlers Danlos syndrome report an increase in adverse events, including cardiovascular events, after COVID-19 illness or vaccination.

**Methods:**

A cross-sectional web-based survey was made available from November 22, 2021, through March 15, 2022. 368 respondents primarily from the United States self-reported data including diagnosis. We used a Cox proportional hazards model with time varying indicators for COVID-19 illness or vaccination in the previous 30 days.

**Results:**

We found a significantly increased rate of new abnormal heart rhythms reported in the 30 days following COVID-19 illness. No additional cardiovascular events were reported after COVID-19 illness. 2.5% of respondents with COVID-19 illness were hospitalized. We did not find a statistically significant increased rate of cardiovascular events in the 30 days following any COVID-19 vaccination dose. Post COVID-19 vaccination, 87.2% of hypermobile Ehlers-Danlos syndrome respondents endorsed an expected adverse event (EAE), and 3.1% reported an emergency department visit/hospitalization, of those who received at least one vaccine dose. Events possibly reflecting exacerbation of orthostasis/dysautonomia were common.

**Conclusion:**

Respondents did not report an increased rate of any cardiovascular events in the 30 days following COVID-19 vaccination; however, those with hypermobile Ehlers-Danlos syndrome experienced a high rate of expected adverse events after vaccination consistent with a high baseline prevalence of similar symptoms. No cardiovascular events other than new abnormal heart rhythms were reported at any point after a COVID-19 illness.

## Introduction

Hypermobile Ehlers-Danlos syndrome (hEDS) is a heritable connective tissue disorder without a clear etiology primarily associated with generalized joint hypermobility and other multisystem comorbidities including pain, fatigue, orthostasis, sleep disturbance, anxiety, gastrointestinal disturbance, and a poorer health-related quality of life [1]. Many of the symptoms expected during a viral illness or after a vaccination (ex: headache, abdominal pain, joint/muscle pain, palpitations, tiredness, diarrhea) are present at higher rates at baseline in those with hEDS compared to the general population [2]. While there are no therapies specific to hEDS, symptoms of hEDS and comorbidities of hEDS can be managed with physical therapy, exercise, lifestyle modification, and medications [3]. It is not known if the increased baseline symptoms translate to more severe symptoms in response to vaccination and illness and if these then result in increased medical resource use that need to be accounted for in the risk/benefit analysis of vaccination for this specific population. In March 2020, COVID-19 was declared a pandemic, and over time patients with SARS-CoV-2 infection were recognized to be at increased risk for a broad range of acute and non-acute cardiovascular manifestations including cerebrovascular events, dysrhythmias, ischemic and non–ischemic heart disease, pericarditis, myocarditis, heart failure, and thromboembolic disease [4]. Furthermore, vaccines for COVID-19, while conferring substantial protection against severe disease and death, have also been linked to an elevated risk of cardiovascular complications, including myocarditis and pericarditis in a small subset of the population [5–7]. Here, we sought to determine whether individuals with hEDS report an increase in CVEs following COVID-19 illness or vaccination and to obtain data on adverse event rates after COVID-19 vaccination in this population.

## Materials and methods

### Study design and setting

This was a cross-sectional web-based survey. The questionnaire was reviewed by experts in connective tissue disorders, allergy, and REDCap questionnaire design. The survey was tested in two rounds with evaluation by approximately 20 individuals not involved in the survey development evaluating the clarity of the questions, the flow of the survey, and the overall user experience. Feedback from the pre-test participants was incorporated to refine the survey.

### Study and source population

A web-based survey was advertised through the Marfan Foundation website and made available from November 22, 2021, through March 15, 2022. Responses were not restricted based on ISP location, although participants were asked if they were located in the United States and questions indicated the study would be asking about vaccines available in the United States. Respondents could enter data for themselves and/or family members. A diagnosis of hypermobile Ehlers-Danlos was self-reported. Parents/guardians were instructed to fill out the survey for participants less than 18 years of age. During the survey period, COVID-19 vaccinations for younger children became available, and parents/guardians were asked to wait at least eight weeks after the second vaccination to fill out the survey for children less than 12 years of age. Inclusion criteria included all patients 18 years old or older listing a diagnosis of hEDS, or those younger than 18 years old with a diagnosis of hEDS who had data entered by a parent or guardian. Exclusion criteria included those listing that the subtype of EDS was unknown, listing additional subtypes of Ehlers-Danlos Syndromes as possible diagnoses, or listing that the diagnosis of hEDS was being considered but not final.

### Data collection tool and procedure

Advertised web address directed respondents to a REDCap [8, 9] based data collection tool into which respondents entered their own data. Respondents were asked to provide information regarding the following: 1) Diagnosis and demographics; 2) date of a COVID-19 positive test/diagnosis and disease severity; 3) dates/type of COVID-19 vaccination received; 4) symptoms and timing/treatment of symptoms after vaccination; 5) occurrence of any cardiovascular event (CVE). Respondents were asked to report CVEs that occurred between January 1, 2019, and the date of survey completion. CVEs were chosen as those reported in the literature as possibly related to COVID illness and vaccination as well as complications seen in connective tissue disorders as defined by a connective tissue disorders genetics expert. CVEs were categorized as new aneurysm; new dissection; vascular rupture; heart inflammation (myocarditis) requiring hospitalization; new abnormal heart rhythm; cardiovascular surgery; death due to a proven cardiovascular event; and sudden unexplained death. Respondents were asked about seventeen post vaccination symptoms. These symptoms were chosen for several reasons. First expected adverse events (EAE) were those symptoms asked during the COVID vaccine trials [10]. EAEs included nausea, vomiting, diarrhea, abdominal cramps; redness/swelling at injection site; fever; chills; fatigue, body and/or muscle aches (myalgia); joint pain (arthralgia); and headache. Given the high rate of allergic reactions reported to COVID vaccination, we also chose to ask subjects about symptoms that might be suggestive of allergic events. These symptoms were chosen to be consistent with the Second National Institute of Allergy and Infectious Disease/Food Allergy and Anaphylaxis Network (NIAID/FAAN) symposium definitions [11]. For analysis these were categorized into symptoms that suggested a possible allergic reaction (SSPAR), and symptoms possibly indicating exacerbation of orthostasis/dysautonomia (EOD), given the susceptibility of those with hEDS to autonomic dysfunction, including postural orthostatic tachycardia syndrome (POTS), vasovagal syncope or neurally mediated hypotension (NMH), orthostatic hypotension (OH), and orthostatic intolerance [12, 13]. SSPARs were itching, hives, rash; swelling of face/lips/eyes/throat/tongue; feeling of throat itching/scratchiness/closing, lump in throat; new hoarseness of voice; runny nose, itchy eyes; coughing spells, wheezing, shortness of breath/difficulty breathing. EOD were palpitations (heart racing), dizziness; passing out/loss of consciousness; low blood pressure/hypotension.

### Data quality control

Entries with no date entered for any event were removed. Participants were asked if they had ever entered data for the survey before. Those answering yes were further manually inspected and in the three cases they could be resolved due to repeated email/phone number and/or multiple exact dates of events. Respondents were asked to provide as much information regarding the date of events as they could recollect. When provided, the exact date was used for analysis. If only the month and year were provided, the date was imputed as the 15th of the month. If only the year was provided, the date was considered missing. If subjects were unable to provide the exact date but provided information that two events occurred in the same month, they were given the opportunity to temporally order the events. Data were excluded from analysis if COVID-19 illness was reported to occur prior to March 1, 2020; an event was specified to occur after the survey completion date; a vaccination was reported to occur prior to December 1, 2020; or two COVID-19 vaccinations were reported on the same day.

### Ethical consideration

All procedures performed in studies involving human participants were in accordance with the ethical standards of the institutional and/or national research committee and with the 1964 Helsinki declaration and its later amendments or comparable ethical standards. This study was approved by the Johns Hopkins institutional review board as exempt research under the DHHS regulations. As this was exempt research no specific consent was required, however the front matter to the survey contained the statement: “Answering the survey questions implies consent for participation in this study”.

### Statistical analysis

To determine if COVID-19 illness or vaccination was associated with an increase in CVEs, we used a Cox proportional hazards model with time varying indicators for COVID-19 illness or vaccination in the previous 30 days. The dependent variable is a CVE. The independent variable is COVID-19 illness or vaccination in the previous 30 days. Evidence of an increase in CVEs in the 30 days following COVID-19 or vaccination was assessed via a Wald test of the estimated effect of each time-varying covariate. The start of the modeling period was January 1, 2019, and participants who did not experience a CVE were censored on the date of survey completion; all participants were censored by March 15, 2022. Hence, each respondent contributed either a CVE date or a censoring time to the data. The Cox model was fit separately for all CVEs, new abnormal heart rhythm, and the group of any CVE other than new abnormal heart rhythm (new aneurysm, new dissection, vascular rupture, myocarditis requiring hospitalization, cardiovascular surgery, or death). Analysis was performed using STATA [14] and R [15].

## Results

There were 376 respondents. Entries without any dates were removed and the data from duplicate submissions for three respondents were merged, leaving data from 368 respondents of sufficient quality for analysis: 17 male, 332 female, and 19 who did not answer or preferred not to say. 357 respondents indicated they were from the United States. Of the eleven indicating they were located outside the United States, seven were vaccinated, six with a vaccine available in the United States and one not answering. The mean age ± standard deviation and range in years for respondents was 38.5 ± 13.1 years (0.04–73.4). Eighty respondents reported a COVID-19 illness (one did not report a date of illness), 78 of whom were treated at home and two of whom were admitted to the hospital. Of the 2 hospitalized patients, 1 required ICU care and neither were intubated. 290 (78.8%) received at least one dose of a COVID-19 vaccine and 176 (47.8%) reported having received three vaccine doses. Of the total 737 vaccine doses received, 38.3% were manufactured by Moderna, 57.9% by Pfizer, 3.1% by Johnson and Johnson and 0.7% by an unknown or unspecified source.

**Fig 1:**
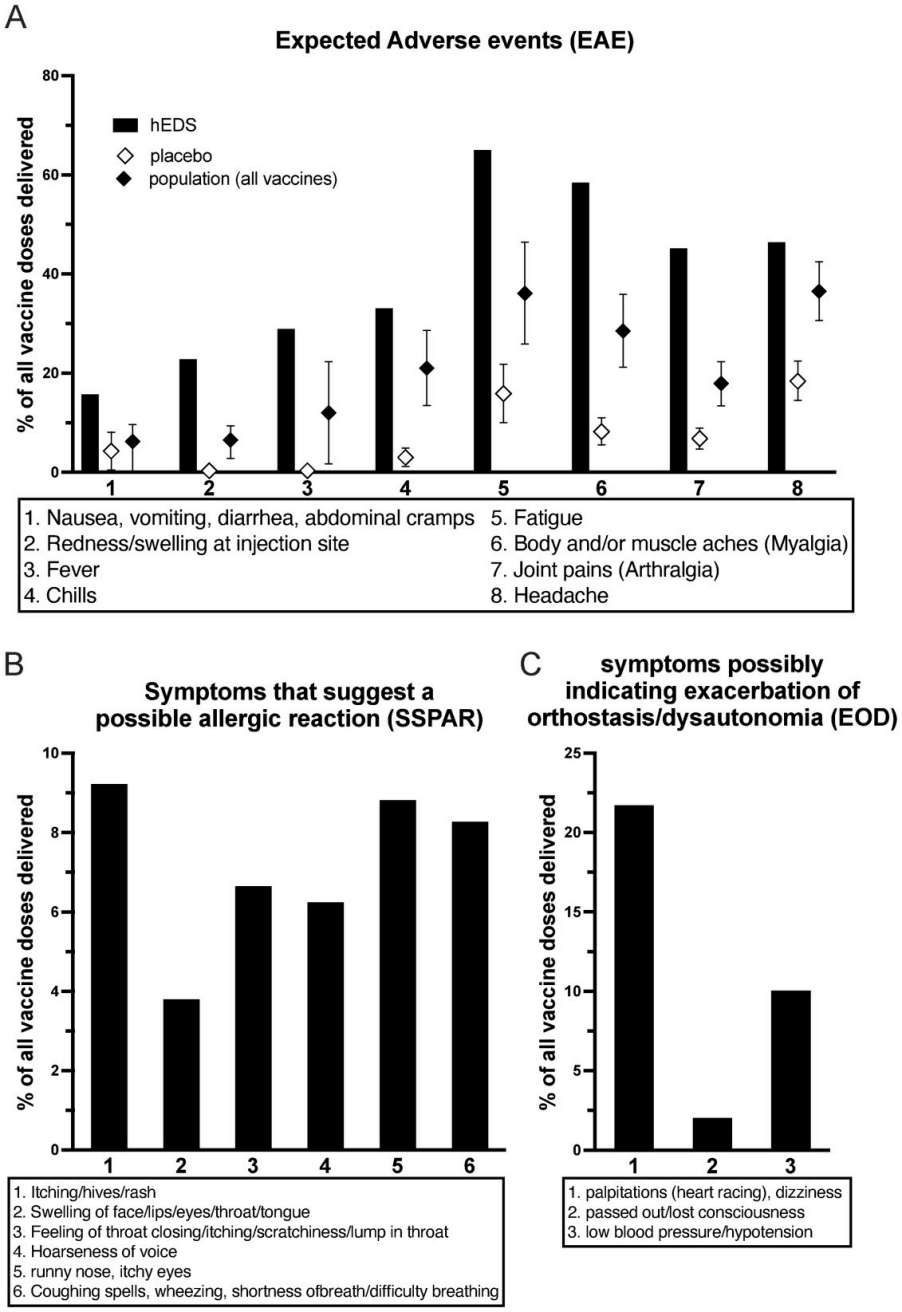
(A) Percent of vaccine doses resulting in an adverse event in respondents compared to the general population as reported in reference 10. (B) Percent of vaccine doses resulting in symptoms suggestive of a possible allergic reaction. (C) Percent of vaccine doses resulting in symptoms possibly indicating exacerbation of orthostasis/dysautonomia.

253/290 (87.2%) respondents who received at least one vaccine dose reported at least one expected adverse event (EAE) following vaccination, with a total of 2326 EAEs reported. 538/737 (73%) of vaccinations were followed by an EAE. Respondents had a higher number of each EAE compared to the general population as shown in Fig 1A [10].

92/290 respondents (31.7%) reported 154 vaccinations (20.9%) with SSPAR (Fig 1B). 102/290 respondents (35.2%) reported 178 vaccinations (24.2%) with EOD (Fig 1C).

11 (1.5%) vaccine doses in 8/290 subjects (2.9%) resulted in an emergency department visit and 3 (0.4%) vaccine doses in 2/290 subjects (0.7%) resulted in a hospitalization. A total of 9/290 subjects (3.1%) reported an emergency department visit or hospitalization after at least 1 vaccine dose. Of the 284 reactions needing treatment, 2 (0.7%) were treated with epinephrine, 25 (8.8%) with steroids, and 26 (9.2%) with nebulizers/inhalers. About a third (32.7%) of those treated received antihistamines and most also used ibuprofen (63%) and/or acetaminophen (53.5%). Most reactions (58.8%) occurred within 6 hours of vaccination.

**Fig 2:**
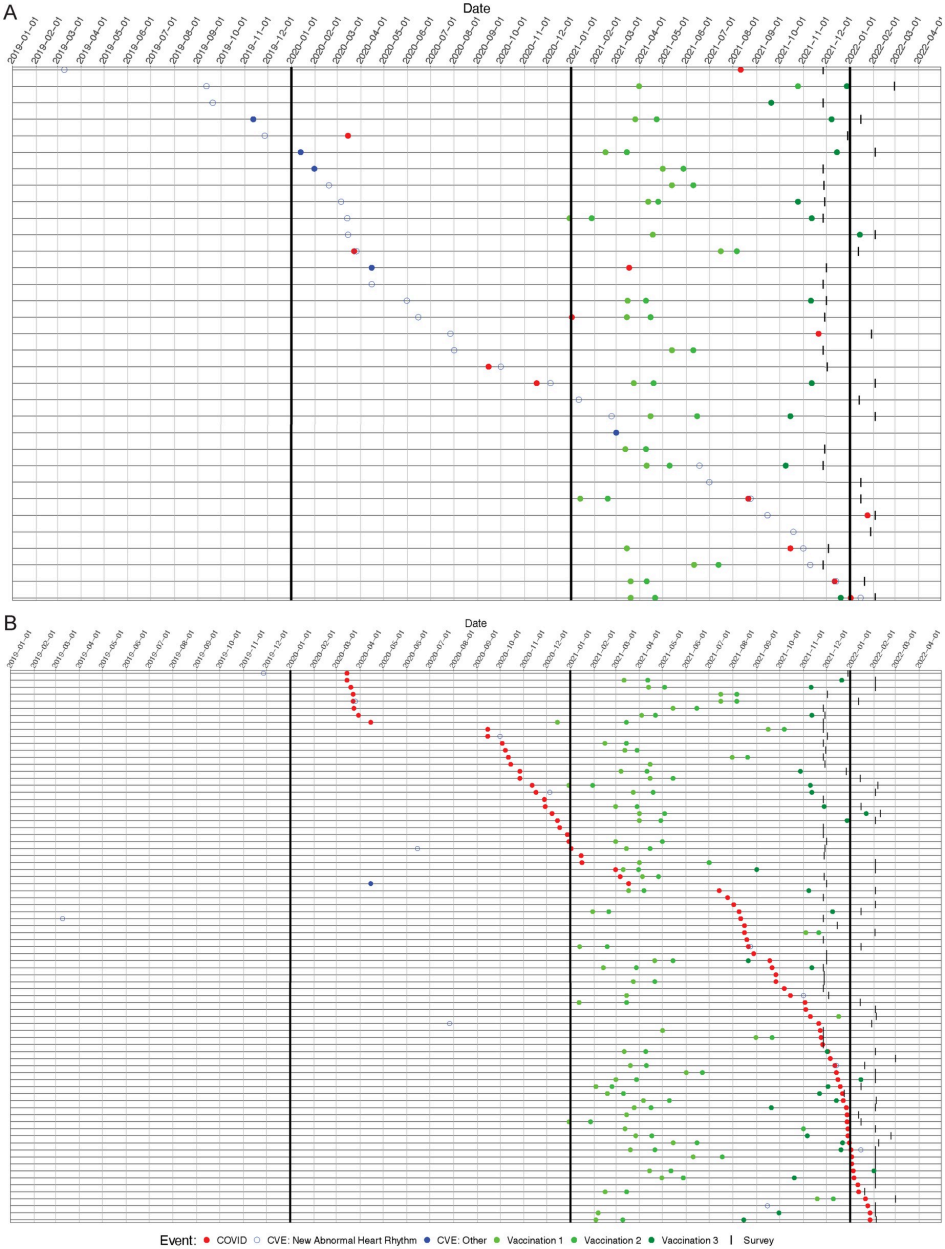
Respondents with (A) a reported CVE (ordered by time of CVE), (B) a reported positive COVID-19 test/diagnosis (ordered by time of COVID-19 positive test/diagnosis). Horizontal lines represent individual subjects, vertical lines separate months, and symbols denote when COVID (red), CVE (open blue: new abnormal heart rhythm; closed blue: other), vaccination (green; light to dark represents doses), and survey completion (tick mark) occurred. For example, the first row of Fig 2A denotes a survey respondent with a CVE (new abnormal heart rhythm) in early March 2019, COVID-19 illness in early August 2021, no vaccine doses, and survey completed in late November 2021. This individual appears in the top line because they had the earliest CVE among the respondents.

We next looked for an association of CVEs with either vaccination or COVID-19 diagnosis. There were 43 CVEs reported in 41 participants during the study period (3 new aneurysms, 1 new dissection, 1 vascular rupture, 3 myocarditis requiring hospitalization, 32 new arrhythmias, and 3 cardiovascular surgeries). 8 of the CVEs occurred prior to March 1, 2020, and 25 CVEs occurred after that time, and 8 were missing dates. We were able to assign an exact date for the CVE for 28 of these patients. Twenty-eight events with a date available were new abnormal heart rhythms. A total of 290 subjects received vaccination dose 1 (229 knowing the exact date and 17 set to missing), 271 received dose 2 (209 knowing the exact date and 27 set to missing), and 176 received dose 3 (149 knowing the exact date and 7 set to missing). 93.9% (31/33) of subjects with a CVE had a vaccination date used for analysis. Occurrences of CVE, COVID-19 diagnosis, vaccination, and survey response are shown in Fig 2 and Tables 1 and 2.

**Table 1:** Summary of CVE events in those with and without a report of a COVID-19 illness.

	New abnormal Heart rhythm	CVE other than new abnormal heart rhythm	No CVE reported	Total
COVID-19 illness reported as yes	12*	1**	66	79
COVID-19 illness reported as no	16	4	268	288
COVID-19 illness unknown (missing)	0	0	1	1
Total	28	5	335	368

*7 of these events occurred in 1 month after COVID

**No such event was reported after COVID-19 illness, although subjects had differing lengths of follow-up after COVID-19 illness

**Table 2:** CVEs reported, categorized by number of vaccine doses.

	New abnormal heart rhythm	CVE other than new abnormal heart rhythm	No CVE reported	Total
3 vaccine doses*	8	2	148	158
2 vaccine doses*	9	1	83	93
1 vaccine dose*	2	0	24	26
0 vaccine doses	9	2	80	91
Total	28	5	335	368

* A vaccine dose is only counted when a date is assigned for that dose. Therefore in the first row (3 vaccine doses), subjects are only included in that row if they had 3 vaccine doses, all of which have a date assigned (either known or imputed to the 15th of the month, and no doses with an unknown date)

Approximately 80% of the reported CVEs were new abnormal heart rhythms. Report of COVID-19 illness was associated with a report of a new abnormal heart rhythm in the following 30 days compared to other time periods (p < 0.001; Table 3 Model 2).

**Table 3:** Cox proportional hazards model results for COVID-19 illness or vaccination and CVEs. There were no cases of CVEs other than new abnormal heart rhythm in the 120 days following vaccination or COVID-19 illness, therefore it was not possible to estimate an association between COVID-19 vaccination or illness and CVEs other than new abnormal heart rhythms.

	Outcome	Risk Factor	HR	Lower 95% CI Bound	Upper 95% CI Bound	p value
Model 1	Time to Any CVE	Any vaccination dose in the past 30 days	1.38	0.28	6.73	0.691
Model 2*	Time to new abnormal heart rhythm	Any vaccination dose in the past 30 days	1.80	0.34	9.60	0.492
		COVID-19 illness in the past 30 days	116.92	35.94	390.21	<0.001

* These variables run as a single model

Vaccination was not found to be statistically significantly associated with an increased rate of reporting a new abnormal heart rhythm within one month after vaccination compared to other time periods (p = 0.49, Table 3 Model 2), nor was it statistically significantly associated with an increased rate of reporting any CVE (when considered as a whole; p = 0.69, Table 3 Model 1). It was not possible to estimate an association between COVID-19 vaccination or illness and CVEs other than new abnormal heart rhythms as only 5 such events were reported by survey respondents, and none were reported after vaccination or illness (Tables 4 and 5).

**Table 4:** Summary of the timing of new abnormal heart rhythms relative to COVID-19 illness and vaccination.

	Number of new abnormal heart rhythms occurring in the listed time period (Number of respondents still being followed at the beginning of time period)				
Time Period	Illness	Vaccination Dose 1	Vaccination Dose 2	Vaccination Dose 3	Any Vaccination
**≥ 120 days after**	**0 (38)**	**5 (260)**	**3 (228)**	**0 (15)**	**5**
**90-<120 days after**	**0 (42)**	**0 (265)**	**1 (232)**	**0 (38)**	**1**
**60-<90 days after**	**0 (51)**	**1 (271)**	**0 (237)**	**0 (81)**	**1**
**30-<60 days after**	**0 (65)**	**0 (273)**	**1 (241)**	**0 (128)**	**1**
**< 30 days after**	**7 (79)**	**1 (273)**	**1 (244)**	**1 (169)**	**2**
> 30 days before	0 (79)	0 (273)	0 (244)	0 (169)	0
30-<60 days before	0 (79)	1 (273)	0 (244)	0 (169)	1
60-<90 days before	0 (79)	0 (273)	0 (244)	0 (169)	0
90-<120 days before	1 (79)	1 (273)	1 (244)	1 (169)	3
≥ 120 days before	4 (79)	9 (273)	9 (244)	8 (169)	12

**Table 5:** Summary of the timing of CVEs other than new abnormal heart rhythms relative to COVID-19 illness and vaccination.

	Number of CVEs other than new abnormal heart rhythms occurring in the listed time period (Number of respondents still being followed at the beginning of time period)				
Time Period	Illness	Vaccination Dose 1	Vaccination Dose 2	Vaccination Dose 3	Any Vaccination
**≥ 120 days after**	**0 (38)**	**0 (260)**	**0 (228)**	**0 (15)**	**0**
**90-<120 days after**	**0 (42)**	**0 (265)**	**0 (232)**	**0 (38)**	**0**
**60-<90 days after**	**0 (51)**	**0 (271)**	**0 (237)**	**0 (81)**	**0**
**30-<60 days after**	**0 (65)**	**0 (273)**	**0 (241)**	**0 (128)**	**0**
**< 30 days after**	**0 (79)**	**0 (273)**	**0 (244)**	**0 (169)**	**0**
> 30 days before	0 (79)	0 (273)	0 (244)	0 (169)	0
30-<60 days before	0 (79)	0 (273)	0 (244)	0 (169)	0
60-<90 days before	0 (79)	0 (273)	0 (244)	0 (169)	0
90-<120 days before	0 (79)	0 (273)	0 (244)	0 (169)	0
≥ 120 days before	1 (79)	3 (273)	3 (244)	2 (169)	3

Bolding indicates time periods of interest in analysis: CVEs that occurred after either COVID-19 illness or vaccination. The number in parentheses under “Illness” refers to the number of subjects reporting COVID-19 illness still being followed at the start of that time period; note this number is not reduced by those who reported a CVE earlier. The number in parentheses under the vaccination dose columns are analogous. For example, there were no reported cases of new abnormal heart rhythm in the 30–60 days following COVID-19 illness, and 65 respondents were still being followed 30 days post COVID-19 illness. In other words, of the 79 total respondents reporting COVID-19 illness, for whom we knew a date of illness, 14 respondents completed the survey within 30 days of reporting COVID-19 illness. Vaccination dose columns are not mutually exclusive. For example, 1 respondent reported a new abnormal heart rhythm within 30 days following their first vaccine dose (all respondents reporting vaccine dose 1, 273, were still being followed at the start of this time period). For that subject, their CVE also occurred in the 30 days following vaccine dose 2. A different subject reported a new abnormal heart rhythm within 30 days of vaccine dose 3. The rows of the “Any Vaccination” column are not mutually exclusive. For example, if a respondent reported a CVE 20 days post vaccine dose 1, 50 days post vaccine dose 2, and 150 days post vaccine dose 3, they would appear in the rows “< 30 days”, “30-<60 days”, and “> = 120 days”. If a subject reported a CVE > 120 days post all 3 doses, they would only be counted once in the “> = 120 days” row.

## Discussion

Both COVID-19 illness and vaccination have been associated with an increase in adverse cardiovascular outcomes. We evaluated self-reported experiences with COVID-19 illness and COVID-19 vaccination in individuals with hEDS. We found that 2/80 respondents reported hospital admission due to COVID-19. Respondents also reported high rates of EAEs after vaccination. These or similar symptoms are all found at higher baseline rates in those with hEDS compared to the general population [2]. We found an increased rate of new abnormal heart rhythms within the 30 days following a COVID-19 illness but not following COVID-19 vaccination. There was not an increase in reports of any CVE after COVID-19 vaccination. It is not possible to discern which dysrhythmias were short-lived and may represent palpitations or situational tachycardia versus indicative of sustained pathology and/or predisposition. While dysrhythmias have been seen in the general population hospitalized with COVID-19 [16], other studies did not find a proarrhythmic effect of COVID-19 illness after resolution of acute illness [17]. As most critical illnesses increase the risk of cardiac arrhythmia, there are no definitive data establishing a virus-specified causal association between COVID-19 and incident arrhythmia [18]. Given that patients with hEDS may have an associated dysautonomia, their risk of COVID-19 associated new arrhythmia may exceed that observed in the general population. Those with hEDS experienced low rates of vascular aneurysm, dissection, or rupture. However, that these events were reported at all by respondents who self-identified as having hEDS suggests that there may be a level of misdiagnosis in this population.

There is a documented but low risk of anaphylactic reaction to COVID-19 vaccinations. Recent analysis estimated a rate of anaphylaxis following COVID-19 mRNA vaccination of 3.29–5.58/million doses [19, 20], compared to a historical estimated anaphylactic rate of 1.3/million doses for other vaccines [21]. Respondents with hEDS had SSPAR in 20.9% of doses received, although sufficient data were not available to categorize cases of anaphylaxis. Given this study’s survey nature, there was no independent verification of the reaction or laboratory measurements (e.g. tryptase levels) obtained.

Lastly as dysautonomia and orthostatic issues are commonly seen in hEDS, we asked about symptoms that might reflect worsening of these symptoms. 21.7% of vaccine doses resulted in palpitations and/or dizziness and 10% in hypotension, and 2% with loss of consciousness. These should be compared to over 80% of those with hEDS reporting dizziness and approximately 50% reporting palpitations in the last 30 days at baseline [2]. Most vaccination reactions occurred within 6 hours and 0.4% of vaccination doses caused symptoms significant enough to result in hospitalization. 3.1% of respondents receiving at least one vaccine dose reported an ED visit or hospitalization post COVID-19 vaccination, and 2.5% of respondents reporting COVID-19 illness were hospitalized.

Study limitations include the exclusive use of self-reported survey data, and our study population did not have balanced gender representation. At the time of the survey, the risk of repeated infection was not fully appreciated and was not assessed. Ascertainment biases are also possible. Some complications, such as a new abnormal heart rhythm, might only be detected once someone is already receiving medical attention due to COVID-19. Mild or asymptomatic cases of COVID-19 could also be underreported, and subjects might be more likely to respond to the survey if they have had more healthcare interactions, such as those with either CVEs or COVID-19 compared to those who had neither. If subjects who experienced both are more likely to complete the survey, this would likely result in an overestimation of the rate of CVE following COVID-19. Furthermore, those analyses where we failed to find associations could be due to limited events.

## Conclusions

Despite these limitations, our data provide reassurance that while patients with hEDS reported an increase in diagnosis of new abnormal rhythms in the 30 days following COVID-19 illness, that among the 80 respondents who reported COVID-19 illness, no CVEs other than new abnormal rhythm was reported at any point after the illness. Respondents did not report an increased rate of any CVE in the 30 days following COVID-19 vaccination; however, those with hEDS experienced a high rate of expected adverse events consistent with a high baseline prevalence of similar symptoms. Likely related, there is a concomitant high rate of emergency department visits and hospitalization following COVID-19 vaccination. It has been suggested that interdisciplinary collaboration is needed to address the biological, psychological, and psychosocial components of symptoms in those with hEDS [22], and this population may benefit from increased pre-vaccination counseling and post-vaccination surveillance and support. We suggest that such support include both anticipatory guidance as well as medical therapies to mitigate predicted symptoms. Further studies are needed to investigate whether such a program might ameliorate symptoms sufficiently to decrease the need for emergency department utilization.

## Supporting information

S1 Fig All survey respondents. Subjects are ordered in the following way: subjects with CVE and COVID, ordered by time of CVE; subjects with CVE only, ordered by time of CVE; subjects with COVID only, ordered by time of COVID, subjects without any CVE or COVID, with at least one dose of vaccine, ordered by time of survey completion. Horizontal lines represent individual subjects, vertical lines separate months, and symbols denote when COVID (red), CVE (open blue: new abnor- mal heart rhythm; closed blue: other), vaccination (green; light to dark represents doses), and survey completion (tick mark) occurred.
(TIF)

## Abbreviations

CVEs: Cardiovascular Events
EAE: Expected Adverse Events
EOD: exacerbation of orthostasis/dysautonomia
hEDS: Hypermobile Ehlers-Danlos Syndrome
SSPAR: Symptoms that suggest a possible allergic reaction
