# Supplementary figures and images for: Web-based survey investigating cardiovascular complications in hypermobile Ehlers-Danlos syndrome after COVID-19 infection and vaccination
Source: PLoS One. 2024 Mar 21;19(3):e0298272. doi: 10.1371/journal.pone.0298272 (PMC10956836; doi:10.1371/journal.pone.0298272)

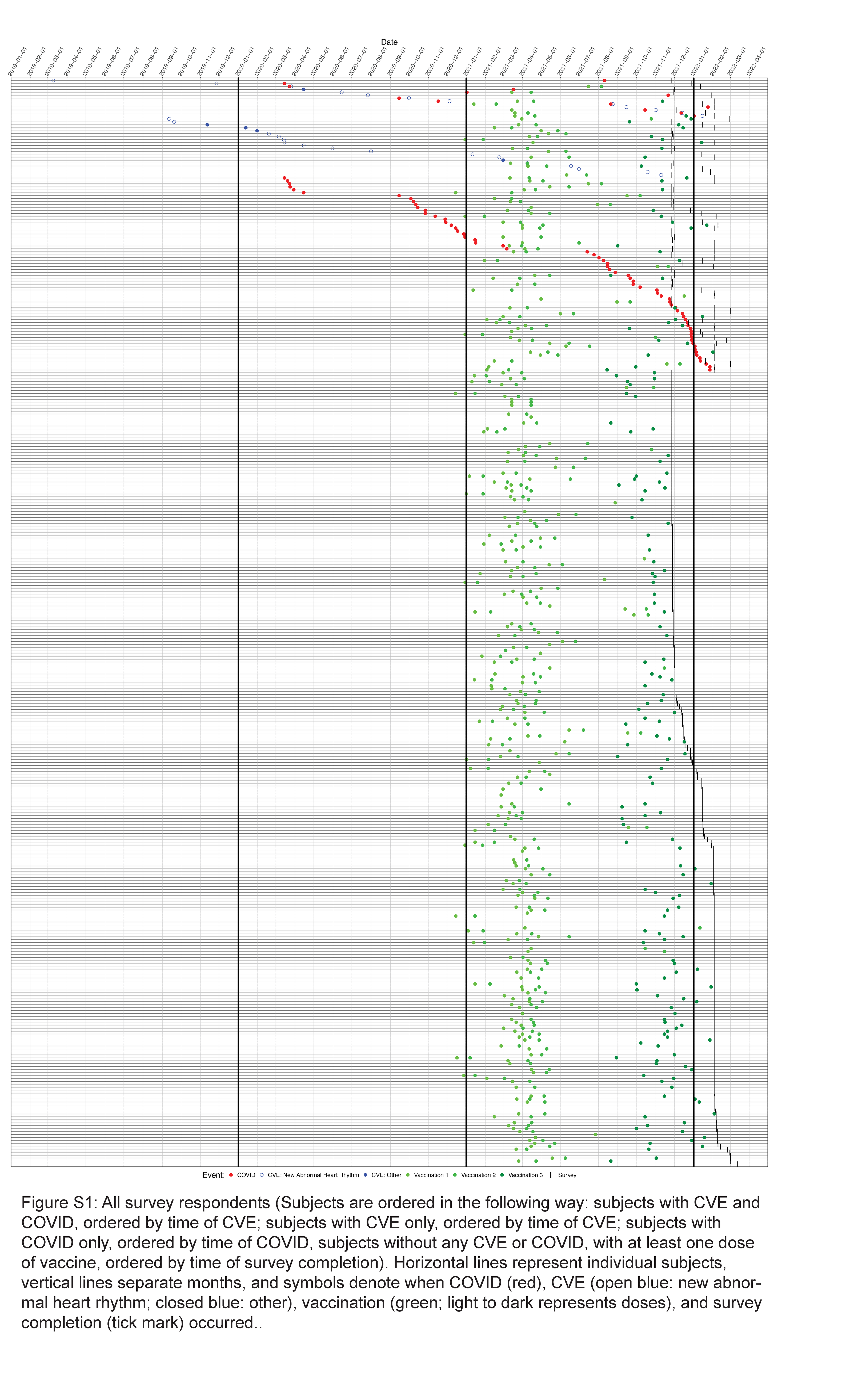

Supplement: S1 Fig — Subjects are ordered in the following way: subjects with CVE and COVID, ordered by time of CVE; subjects with CVE only, ordered by time of CVE; subjects with COVID only, ordered by time of COVID, subjects without any CVE or COVID, with at least one dose of vaccine, ordered by time of survey completion. Horizontal lines represent individual subjects, vertical lines separate months, and symbols denote when COVID (red), CVE (open blue: new abnor- mal heart rhythm; closed blue: other), vaccination (green; light to dark represents doses), and survey completion (tick mark) occurred. (TIF) [file pone.0298272.s001.tif]
